# Future methane emissions from lakes and reservoirs

**DOI:** 10.1038/s44221-025-00532-6

**Published:** 2025-11-04

**Authors:** David Bastviken, Matthew S. Johnson

**Affiliations:** 1 Department of Thematic Studies–Environmental Change, Linköping University, Linköping, Sweden; 2 Earth Science Division, NASA Ames Research Center, Moffett Field, CA USA

**Keywords:** Limnology, Biogeochemistry, Environmental impact

## Abstract

Global lake and reservoir water surfaces were recently estimated to contribute ~10% of global methane (CH_4_) emissions. The sensitivity of these emissions to climate and environmental change is a growing concern. Here we present data-driven, globally gridded modelling of future open-water CH_4_ fluxes under different scenarios. We included multiple potential predictor variables and available peer-reviewed flux data focusing on in situ-verified relationships. The results indicate total lake and reservoir CH_4_ emissions increases of 24–91% under the IPCC Shared Socioeconomic Pathway (SSP) climate change scenarios SSP1-2.6 to SSP5-8.5 by 2080–2099. Effects of changed temperature and seasonality dominated these increases. Area and nutrient load changes also contributed substantially to reservoir emissions. Large absolute changes were predicted at all latitudes. The results demonstrate the urgency in minimizing climate change to avoid substantially increased future inland water CH_4_ emissions.

## Abstract (web summary)

This study uses data-driven modelling to predict a 24–91% increase in methane emissions from global lakes and reservoirs by 2080–2099 under various climate scenarios. Temperature and seasonality changes are key drivers, highlighting the need for climate action.

## Main

Emissions of methane (CH_4_) have increased from 1750 to 2019 contributing a total radiative forcing (direct and indirect) of 0.95 W m^−2^ (refs. ^1,2^). This is 22% of the total radiative forcing of all greenhouse gases (GHGs) and 30.5% of the total radiative forcing from carbon dioxide (CO_2_), CH_4_ and nitrous oxide (N_2_O)^1,2^. Overall, since 1750, CH_4_ levels have increased by a factor of 2.6 (compared to 1.5 for CO_2_ and 1.2 and N_2_O)^2^. Whereas atmospheric increases of CO_2_ and N_2_O have followed a regular trajectory aligned with human activity and fossil fuel use, the increase of atmospheric CH_4_ has much larger interannual- and decadal-variability for yet unknown reasons^3,4^.

CH_4_ has a 20-year global warming potential of 81 ± 26 compared to CO_2_ on a mass basis, but a relatively short atmospheric lifetime (~11 years) (ref. ^2^). Accordingly, increases or decreases in CH_4_ emissions can rapidly and substantially enhance or dampen global warming, making CH_4_ emissions regulation important for the future climate. Possible climate change effects on CH_4_ sources are therefore a concern, and it was recently suggested that the climate sensitivity of CH_4_ emissions is much greater than previously estimated^5^. However, the quantitative magnitudes and drivers of many of these effects remain uncertain, making it challenging to understand past atmospheric CH_4_ concentration variability and accurately forecast CH_4_ emissions into the future.

Open-water surfaces of freshwater lakes and reservoirs collectively represent one of the largest contemporary CH_4_ sources. On a global scale, the water surfaces of lakes and reservoirs have recently been estimated to emit 24–150 and 10–20 Tg CH_4_ yr^−1^, respectively^6–12^. A main CH_4_ source is microbial degradation of organic matter in anoxic sediments^13^. When CH_4_ production rates exceed dissolution, bubbles are formed which can be released and rapidly rise through the water column to the atmosphere—an emissions pathway referred to as ebullition, which often dominates total emissions from open-water environments^7,8^. The CH_4_ getting dissolved in the water follows a slower route towards the atmosphere via advection or turbulent diffusion. A large proportion of this dissolved CH_4_ is consumed by microbial oxidation when reaching portions of the water column where dissolved oxygen (O_2_) or other suitable electron acceptors are available. The remaining dissolved CH_4_ eventually passing the boundary layer at the water–atmosphere interface is emitted by diffusive flux. Methane being formed in oxic surface waters can also add to the diffusive flux^14^. Notably, ebullition and diffusive flux are regulated in different ways as described in detail elsewhere^13,15^.

Methane emissions from lake and reservoir water surfaces can be influenced by environmental change in multiple ways. Microbial CH_4_ production seems more sensitive to temperature than many other metabolic processes such as photosynthesis, oxic respiration and CH_4_ oxidation and increases exponentially with temperature across lake and reservoir types at all latitudes^16,17^. Similar exponential temperature responses have been observed for ebullition^18^ and for total CH_4_ emissions^17,19^. In addition, the length of the ice-free period influences high-latitude lake CH_4_ emissions^20–23^. Increased availability of labile substrates for methanogenesis, by, for example, biological productivity or access to organic matter released from melting permafrost soils, also enhance CH_4_ production^24–26^. Accordingly, changes in lake and reservoir inputs of catchment organic matter or nutrients can affect CH_4_ production rates and emissions. In addition, possible changes in water surface area will be important for overall emissions.

Constraining contemporary global lake and reservoir CH_4_ emissions and extrapolating contemporary assessments to future climate scenarios has been challenging due to limitations in the amount and comparability of flux observations and to the complexity in the regulation of all relevant processes. A data-driven extrapolation approach was recently published to improve contemporary lake and reservoir flux estimates^7,8^. This approach was further developed in this study to predict global lake and reservoir CH_4_ emissions over 20-year periods until the end of the century (2080–2099). It is based on a gridded lake and reservoir area distribution and considers temperature effects on CH_4_ fluxes under established climate change scenarios, along with the effects of ice-free period length, diel flux variability, water body types and ecoclimatic regions. Different flux types, including ebullition, diffusive flux and enhanced diffusive flux in lakes following ice melt and seasonal water column turnover when deep CH_4_-enriched water reaches the surface, were also included. All information used is linked to in situ flux observations integrating the complex interactions of relevant ecosystem processes. Flux data from 767 systems were used. The assessment of future fluxes also considers effects of estimated regional changes in water surface area and in phosphorous levels to account for nutrient load and associated changes in primary productivity.

## Already-large-lake and reservoir CH_4_ emissions may double

**Fig. 1:**
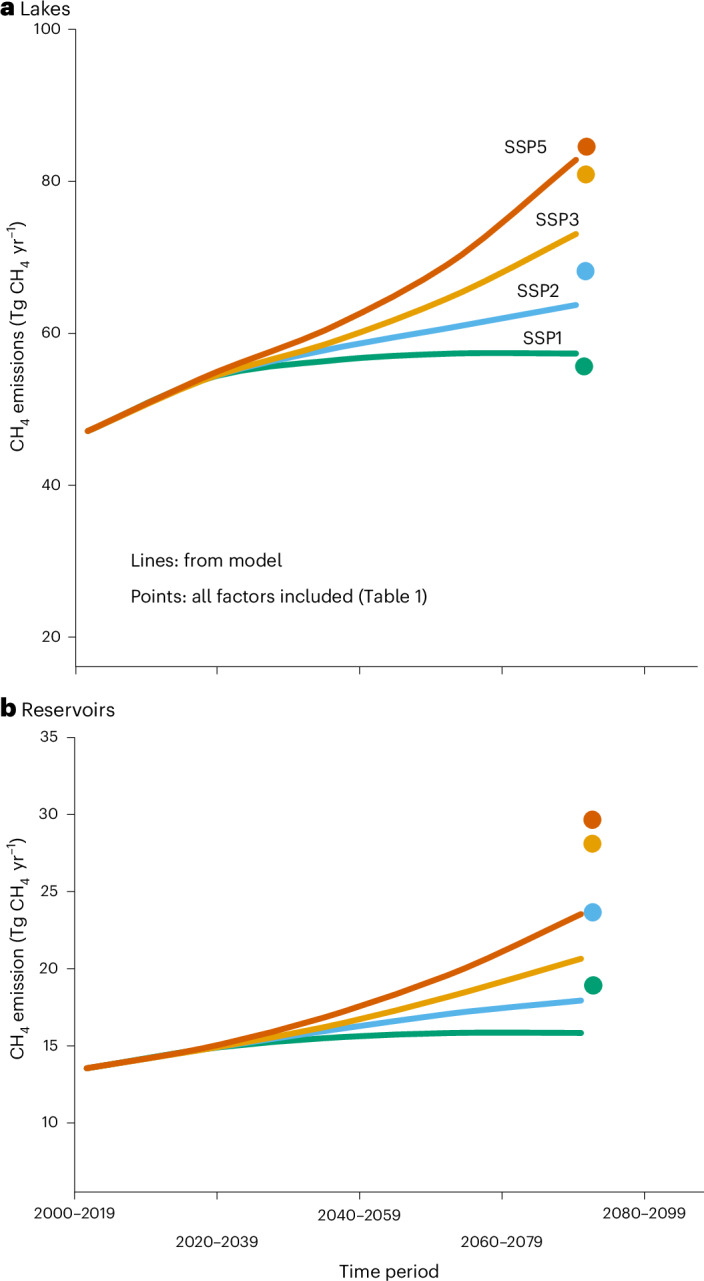
Modelled lake and reservoir CH_4_ emissions over time under different climate scenarios. **a**,**b**, Modelled lake (**a**) and reservoir (**b**) CH_4_ emissions over time under different climate change scenarios (SSP1, SSP2, SSP3 and SSP5). Lines were derived from the model considering primarily the direct temperature response and ice-free period length. The dots represent the expected emissions by the end of the century combining this model and all other change factors in Table 1. Uncertainty estimates and analysis are available in Fig. 5 and Methods. Note that the *y*-axis scales do not start at zero.

**Extended Data Fig. 1:**
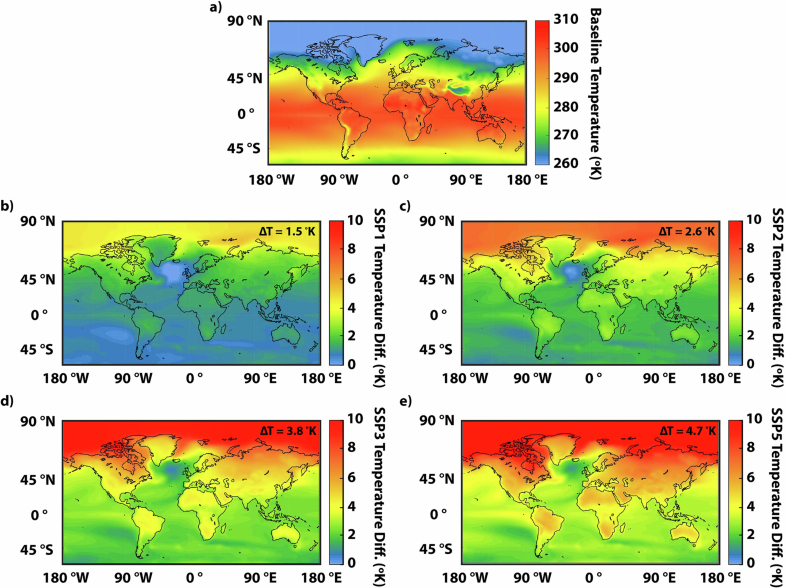
Geospatial distribution of temperatures in different climate scenarios. Extended Data Fig. 1. Geospatial distribution of contemporary (2000-2019) yearly mean 2-metre air temperature from the five CMIP6 model ensemble by grid cell (**a**), and the predicted change by 2080-2099 under SSP1-2.6 (**b**), SSP2-4.5 (**c**), SSP3-7.0 (**c**) and SSP5-8.5 (**d**) climate scenarios using the 20 model ensemble described in Extended Data Table 5. Global mean temperature changes are denoted in the upper right of panels **b**), **c**), **d**), and **e**).

The Shared Socioeconomic Pathway (SSP) climate change scenarios SSP1-2.6 and SSP2-4.5 translates to effective societal transformation and estimated global annual mean temperature increases of 1.5 to 2.6 °C by year 2080–2099 (Extended Data Fig. 1)^27^. Under these scenarios, the combined lake and reservoir water surface CH_4_ emissions were estimated to increase 24–52% in total (from 59 to 73–90 Tg CH_4 _yr^−1^; Table 1 and Fig. 1). The SSP3-7.0 and SSP5-8.5 climate scenarios represent societal inability to mitigate GHG emissions and a resulting annual global mean temperature increase of 3.8–4.7 °C by the end of the century. Under these warmer scenarios, combined lake and reservoir emissions increased 80–91% to 107–112 Tg CH_4_ yr^−1^ (Table 1 and Fig. 1). A change from 59 to 113 Tg CH_4_ yr^−1^, that is, 53 Tg CH_4_ yr^−1^, represents almost a doubling of the lake and reservoir emissions and an ~10% increase of the total global contemporary CH_4_ emissions^3^. An increase of 53 Tg CH_4 _yr^−1^ can also be viewed in the context of the total estimated contemporary emissions from landfills and waste, coal mining or oil and fossil gas emissions (55–71, 23–40 and 60–97 Tg CH_4 _yr^−1^, respectively).

**Table 1:** Estimated present, 2000–2019, and end of the century, 2080–2099, CH_4_ emissions under different climate change scenarios from lakes and reservoirs by ecoclimatic region, lake or flux type, considering results from the multi-factor model and changes in water surface area and nutrient levels

Region, water type or flux type	Area	Present emissions	Results from gridded multi-factor effect model				Area *	Nutrient levels*				Total predicted flux (2080–2099)							
	×10^3^ km^2^	(Tg yr^−1^)	Change factor				Change factor	Change factor				Emissions (Tg yr^−1^)				Change factor			
			1-2.6	2-4.5	3-7.0	5-8.5		1-2.6	2-4.5	3-7.0	5-8.5	1-2.6	2-4.5	3-7.0	5-8.5	1-2.6	2-4.5	3-7.0	5-8.5
**Lakes**																			
Total Boreal–arctic	1,606	14.6	1.31	1.43	1.60	1.81	1.02	0.90	0.97	1.03	0.95	17.0	20.2	23.6	24.7	1.16	1.38	1.62	1.69
Thermokarst	360	3.6	1.44	1.61	1.86	2.19	0.91	0.90	0.97	1.03	0.95	4.2	5.1	6.3	6.8	1.18	1.43	1.74	1.90
Glacial/postglacial	398	2.0	1.30	1.45	1.70	1.95	1.02	0.90	0.97	1.03	0.95	2.4	2.9	3.6	3.8	1.19	1.44	1.78	1.89
Peat pond	97	1.8	1.28	1.44	1.61	1.83	1.02	0.90	0.97	1.03	0.95	2.1	2.6	3.0	3.2	1.17	1.44	1.68	1.77
Organic	67	1.3	1.31	1.31	1.46	1.62	1.02	0.90	0.97	1.03	0.95	1.6	1.7	2.0	2.0	1.20	1.30	1.53	1.56
Other boreal	684	5.9	1.24	1.34	1.42	1.56	1.02	0.90	0.97	1.03	0.95	6.7	7.8	8.8	8.9	1.13	1.33	1.49	1.51
Temperate	1,117	11.4	1.30	1.51	1.87	2.24	1.02	0.90	0.97	1.03	0.95	13.5	17.1	22.3	24.7	1.19	1.50	1.95	2.16
Tropical–subtropical	376	20.0	1.12	1.23	1.37	1.49	1.02	1.07	1.18	1.19	1.12	24.4	29.4	33.2	33.9	1.22	1.47	1.66	1.70
By flux type:																			
*F*_diff_ + *F*_ebul_		40.2	1.26	1.43	1.66	1.92													
Ice out + spring turnover flux		4.5	0.87	0.80	0.73	0.64													
Fall turnover		1.3	1.15	1.23	1.31	1.38													
All lakes	3,099	46.0	1.22	1.36	1.56	1.78						54.9	66.7	79.1	83.3	1.19	1.45	1.72	1.81
**Reservoir open-water fluxes**																			
Boreal–arctic	66	0.4	1.25	1.50	1.75	2.25	1.263	0.90	0.97	1.03	0.95	0.6	0.7	0.9	1.1	1.42	1.85	2.26	2.70
Temperate	124	7.9	1.19	1.37	1.61	1.85	1.263	0.90	0.97	1.03	0.95	10.6	13.3	16.4	17.5	1.35	1.68	2.08	2.21
Tropical–subtropical	107	4.7	1.15	1.28	1.43	1.60	1.263	1.07	1.18	1.19	1.12	7.3	8.9	10.1	10.6	1.56	1.90	2.15	2.25
All reservoirs	297	13	1.18	1.34	1.55	1.77						18.5	23.0	27.4	29.1	1.43	1.77	2.11	2.24
**Lakes and reservoirs**	3,396	59	1.16	1.27	1.44	1.61						73.4	89.6	106.5	112.4	1.24	1.52	1.80	1.91

*Area and nutrient level change estimates were considered outside the gridded multi-factor model at regional resolution for the end-of-century using change factors (Methods).

The change factors represent multipliers by which present fluxes are multiplied to yield predicted effects and can be used to compare effect sizes among predictors. (A change factor of 1.50 corresponds to a 50% increase.) The change factors thereby express relative change that is less sensitive to uncertainty in absolute flux levels and the change factors can be used for comparisons among predictions independently of the absolute flux results. The different climate change scenarios used are denoted by the SSP numbers, that is, 1-2.6, 2-4.5, 3-7.0 and 5-8.5. Uncertainty information is available in main text, Methods and Fig. 5.

**Fig. 2:**
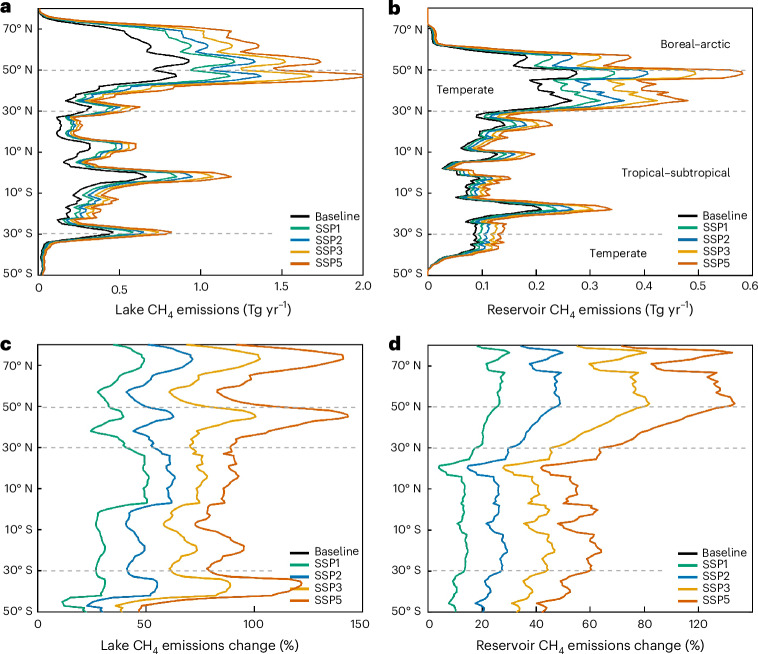
Present-day baseline and predicted future lake and reservoir CH_4_ emissions and relative flux changes by latitude for different climate change scenarios. **a**–**d**, Present-day baseline and predicted future lake and reservoir emissions (**a**,**b**) and percent flux changes (**c**,**d**) by latitude for different SSP climate change scenarios. Dashed grey lines represent approximate latitude transitions between the boreal–arctic, temperate and tropical–subtropical ecoclimatic regions. Text provides details.

**Extended Data Fig. 2:**
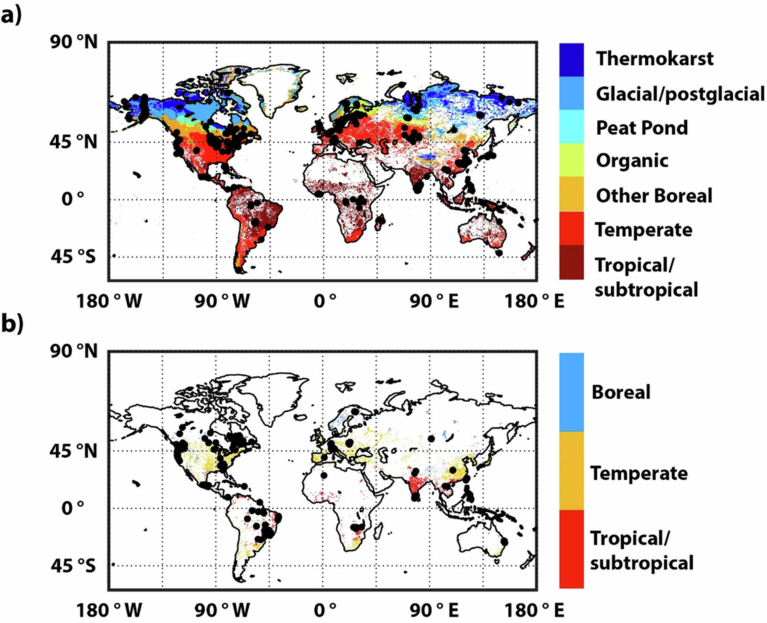
Geospatial distribution of lakes, reservoirs and flux measurements. Extended Data Fig. 2. The geospatial distribution of (**a**) lakes and (**b**) reservoirs in different ecoclimatic regions, and locations of the flux measurements included in the analysis (black spots). Please note that the color bars differ between panels.

Reservoir emissions increased proportionally more than lake emissions (124% versus 81% increase) and contributed 26% of the total combined absolute emissions increase in the SSP5-8.5 climate scenario (Table 1). These patterns reflect the fact that lakes and reservoirs differ in their global areas and geospatial distribution and therefore also in predicted temperature, area and nutrient load changes (Fig. 2, Extended Data Fig. 2 and Methods).

Temperature-related climate effects were most important for the predicted future fluxes (Table 1). Changes in water surface areas and nutrient load modulated the predicted fluxes for lakes but jointly had a larger effect on reservoir emissions. For example, the planned increase in reservoir area had similar effects as the temperature increase in the SSP1-2.6 and SSP2-4.5 scenarios. A lower estimated nutrient load in the SSP5-8.5 scenario compared to SSP3-7.0^28^, made total predicted emissions under these two scenarios more similar than among other climate scenarios (Table 1).

## Effects among different ecoclimatic regions

**Fig. 3:**
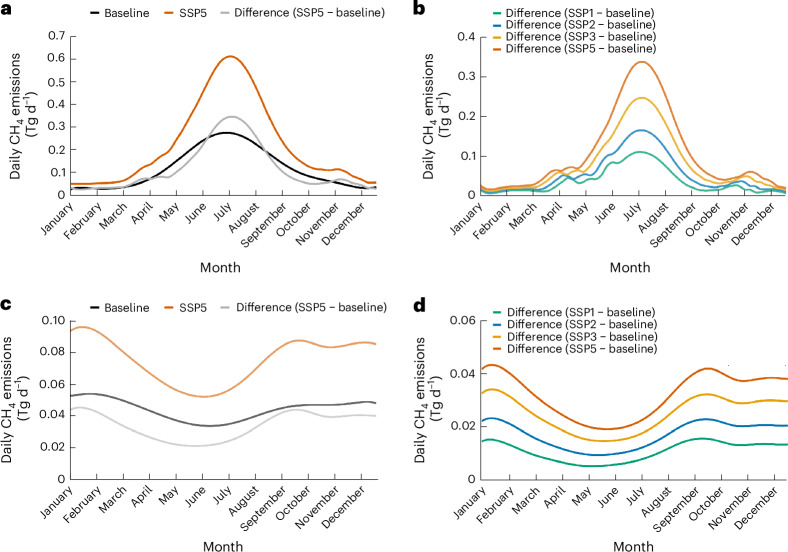
Daily combined global lake and reservoir fluxes under different climate scenarios. **a**–**d**, The 2000–2019 baseline emissions versus predicted emissions under the SSP5-8.5 climate scenario at 2080–2099 (**a**,**c**) and the difference between the two (**b,d**) in the northern and southern hemispheres, respectively. The differences between all used SSP scenarios and the baseline are shown in panel **b** (northern hemisphere) and **d** (southern hemisphere). The irregular patterns in the spring (March–June) and fall/winter (October–December), particularly in the northern hemisphere (**a**,**b**) are due to changes in ice-free season length and timing of emissions upon ice melt and seasonal water column turnover. The smoother portions of the predicted increases between these times primarily reflects temperature-related increases.

The longer ice-free seasons in the future accounted for 9–33% and 14–17% of the temperature effect in the boreal–arctic lake and reservoir CH_4_ emissions, respectively, whereas direct temperature-sensitivity effects dominated the climate-induced flux increase (Extended Data Table 1). Shorter durations of ice cover reduce the time for CH_4_ accumulation in bottom waters during winter at high latitudes. Accordingly, emissions related to ice out and spring water column turnover are predicted to decrease (Extended Data Table 2). Correspondingly, the warmer summers and longer ice-free periods at northern temperate to boreal–arctic latitudes influenced the seasonal pattern of the total emissions (Table 1 and Fig. 3)^7,8^.

**Fig. 4:**
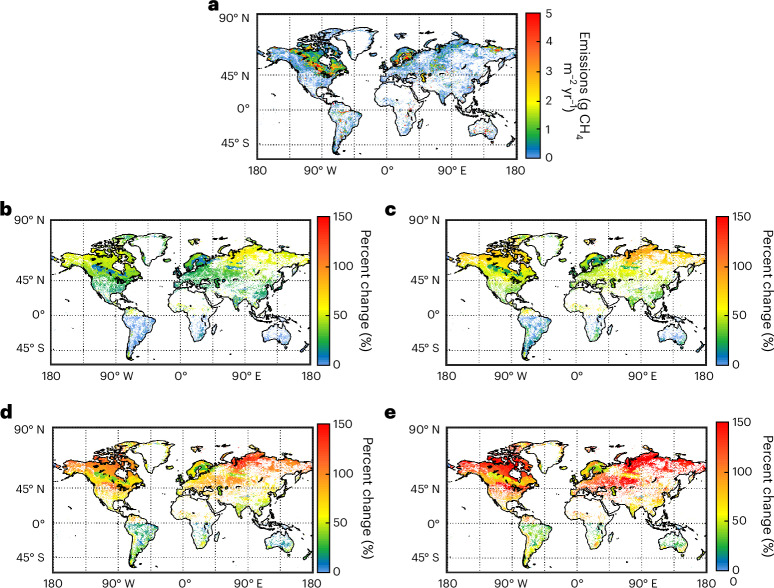
Geospatial distribution of contemporary lake and reservoir emissions and the change in emissions by 2080–2099 under various climate scenarios. **a**–**d**, Geospatial distribution of contemporary (2000–2019) lake and reservoir emissions per m^2^ by grid cell (**a**) and the percent change in emissions by 2080–2099 under the SSP1-2.6 (**b**), SSP2-4.5 (**c**), SSP3-7.0 (**c**) and SSP5-8.5 (**d**) climate scenarios using the 20-model ensemble described in Extended Data Table 5.

The predicted relative increase in emissions due to temperature-related effects was greater at high latitudes for both lakes and reservoirs in all climate scenarios (Fig. 2, Fig. 4 and Table 1). This is expected because of the combined effect of increasing temperatures, prolonged ice-free seasons length (Extended Data Table 2) and the large lake surface area in the temperate to arctic regions (Extended Data Fig. 2). Yet, the tropical and subtropical regions were found to contribute large absolute flux changes in spite of smaller water surface area and lower predicted mean temperature increase (Figs. 2 and 4, Table 1, Extended Data Figs. 1). This illustrates the great importance of high year-round emissions and of the exponential response to increasing temperatures, yielding greater absolute effects per degree of change at higher starting temperatures^16,29^. Accordingly, analyses of relative and absolute changes can yield contrasting patterns, and climate effects in areas with limited relative temperature changes can still be large in absolute terms. Therefore, consideration of climate effects on lake and reservoir CH_4_ emissions at all latitudes is important.

## Discussion

### Emissions uncertainty

This section focuses on uncertainty of the results in relation to the influencing environmental factors and limitations to what environmental change could be considered at this stage. Fundamental uncertainty and limitations associated with the design of our prediction approach are discussed further in the Methods.

**Fig. 5:**
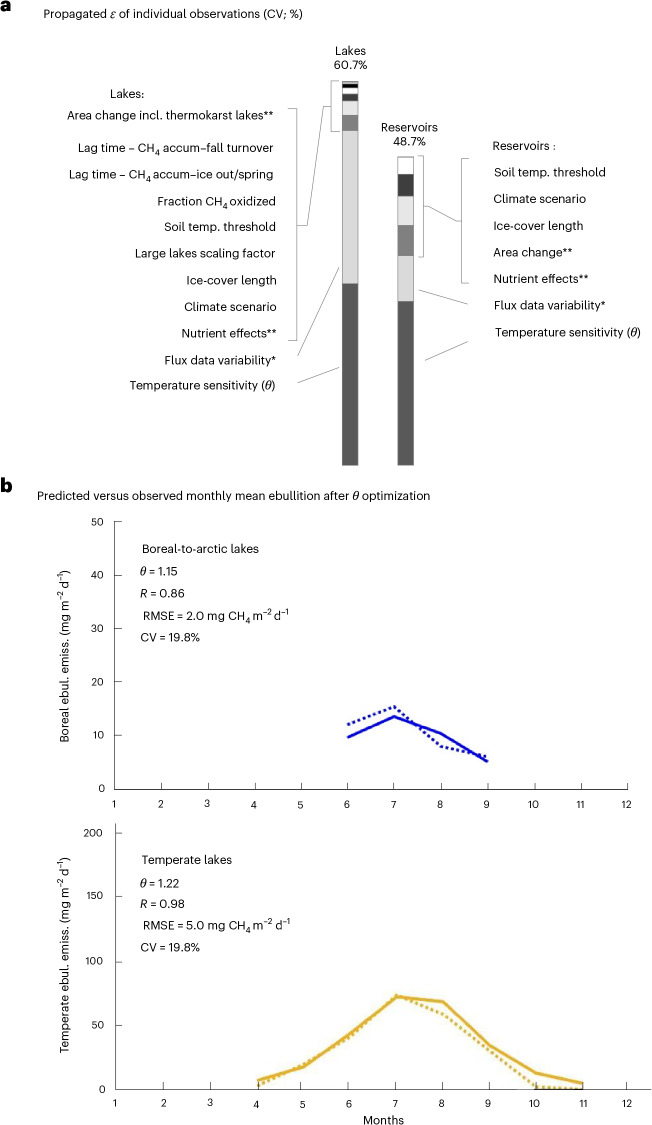
Illustration of two different uncertainty analyses. **a**, Classical propagations of ε expressed as coefficient of variation (CV) of all considered factors (Methods). Such uncertainty propagation reflects uncertainty of the original observations but do not fully account for the integrated total uncertainty, which is often lower as some of the real inherent single-observation variability cancel out when many observations are integrated. Hence, the total uncertainty in **a** represents an upper bound and a likely overestimate, but this approach is useful for comparing the relative uncertainty among considered environmental factors. Note that the inherent uncertainty of some factors has limited influence on relative change with climate scenarios (text). The notes in the panel refer to: *High inherent variability; episodic extreme fluxes are key for the total flux. **Considered outside model by change factors (Table 1 and Extended Data Figs. 3 and 4). **b**, The uncertainty could be constrained by optimizing modelled *θ* for boreal–arctic and temperate lake ebullition (solid line) to monthly averaged in situ observed ebullition (dashed lines). After this optimization, *θ* is informed by the numerous distributed in situ observations and not only *θ* studies in a few systems (Extended Data Table 3). Importantly, this generated considerably lower integrated uncertainty (CV ~20%) while also being consistent with the range of previous single system *θ* observations (Extended Data Table 3; Methods). Hence, while **a** is primarily intended to provide an upper uncertainty bound and a relative comparison among considered environmental predictors, our best use of available information yields a lower overall uncertainty (CV ~20% for temperate and boreal-to-arctic lakes) (**b**). incl., including; accum, accumulation; temp., temperature; ebul. emiss., ebullitive emission; RMSE, root mean square error. ‘Soil temp. threshold’ refers to uncertainty in the definitions of the ecoclimatic regions (Methods).

The total propagated uncertainty for all factors considered and linked to individual observations (*ε*; Methods) corresponded to a coefficient of variation (CV) of 60.7 and 48.7% for lakes and reservoirs, respectively (Fig. 5a). Among the influencing factors considered, the greatest uncertainty was associated with the temperature sensitivity, first assessed from observations in a range of individual systems (Extended Data Table 3). The exponential nature of this relationship makes the choice of temperature sensitivity factor, *θ*, highly important for the results. To constrain this uncertainty, we fitted regional *θ* to multi-system monthly mean fluxes observed at temperate to arctic latitudes. These regional multi-system calibrations resulted in close correspondence between predicted versus observed fluxes and substantially reduced the overall uncertainty (to a CV of 20%; Fig. 5b and Methods), while staying consistent with literature *θ* estimates from individual systems. This validation of single system temperature sensitivity to large amounts of in situ observations across regions represented an important novel way to constrain the prediction uncertainty. The use of a low-end *θ* value for tropical–subtropical areas (close to the minimum *θ* observed; Methods) means that low-latitude temperature sensitivity could be underestimated and makes the low-latitude results conservative.

The variability in the original flux data contributed the second largest uncertainty (Fig. 5a). Because episodic, semi-random, and positive outlier fluxes (ebullition and seasonal outgassing) often dominate cumulative yearly CH_4_ emissions from water surfaces, a large inherent (aleatoric) variability must always be expected. Hence, a low apparent measurement variability can indicate incomplete characterization of the system, while high variability among individual observations is logical if all flux types are represented. Importantly, this type of inherent variability reflects the processes behind the fluxes and is more important for the uncertainty of absolute local flux quantification than for the mean relative regional flux change.

The effects of changes in nutrient load generated the third-largest uncertainty (Fig. 5). The impact of such nutrient changes on the CH_4_ fluxes was larger for reservoirs than lakes due to proportionally larger high-emitting reservoir area in warm regions projected for greatest nutrient load increases. However, the flux uncertainty linked to nutrient load uncertainty was comparatively small and therefore not decisive for the results (Fig. 5a). This also applied for the uncertainty contributions from the remaining factors considered.

Some aspects of future lake and reservoir CH_4_ emissions could not be reliably considered at this stage because of in situ data limitations. One such aspect is the possibility of increased establishment of farm ponds and freshwater aquaculture ponds that have been highlighted as hotspots for CH_4_ emissions^30,31^. It is likely that a future increase in such pond areas would trigger greater emissions. At present, CH_4_ emissions from agricultural ponds in the United States and Australia, and from aquaculture in China, have been estimated to be ~0.17 and 1.6 Tg CH_4_ yr^−1^, respectively^31,32^, indicating a combined potential global emission contribution on the order of 10 Tg CH_4_ yr^−1^. However, the lack of accurate global area estimates for such ponds prevent precise flux estimations and thereby also reliable predictions.

In addition, reservoir turbine degassing is important for total reservoir emissions, but is more related with reservoir design than environmental change and was therefore not included in our estimate. Reservoir turbine degassing has been estimated to increase by 53% from 2010 to 2040^33^, that is, in the order of 5 Tg CH_4_ yr^−1^, which is a small share of the total estimated increase but substantial from the reservoir perspective.

Further, over long timescales, indirect effects may lead to changes in the distribution of the ecoclimatic regions. One example would be the replacement of tundra landscapes by the northward migration of the coniferous taiga forests. This would lead to more lakes being situated in warmer regions and receive more organic matter from the catchments, which would probably lead to increased lake CH_4_ emissions^34^. Over long timescales, transitions from lakes to wetlands with emergent vegetation can also increase CH_4_ emissions via macrophyte gas exchange^35–37^. Such long-term effects are beyond the scope of this study, but in the foreseeable scenarios, the most likely outcome seems to be increasing CH_4_ emissions in the humid biomes. Hence, future studies on effects of landscape development are important to better understand long-term CH_4_ flux changes.

### Directional versus quantitative predictions

**Extended Data Fig. 3:**
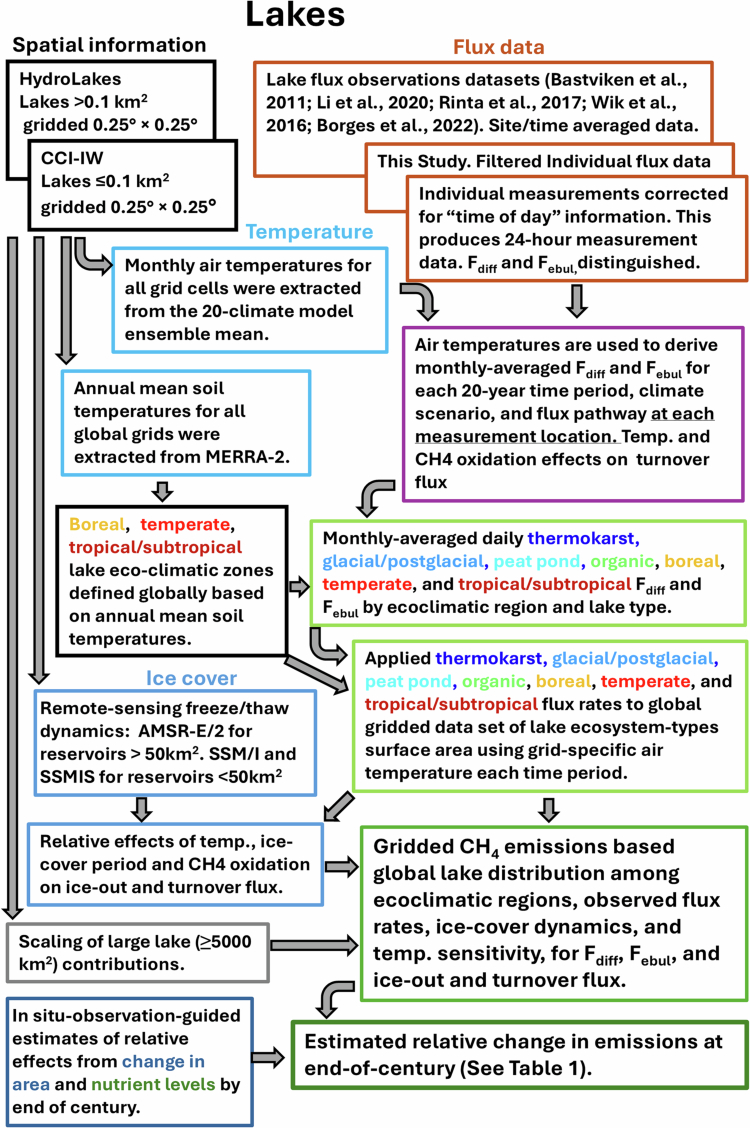
Workflow for the lake emissions. Extended Data Fig. 3. Workflow for the lake emissions, illustrating the various components considered. See Methods for details.

**Extended Data Fig. 4:**
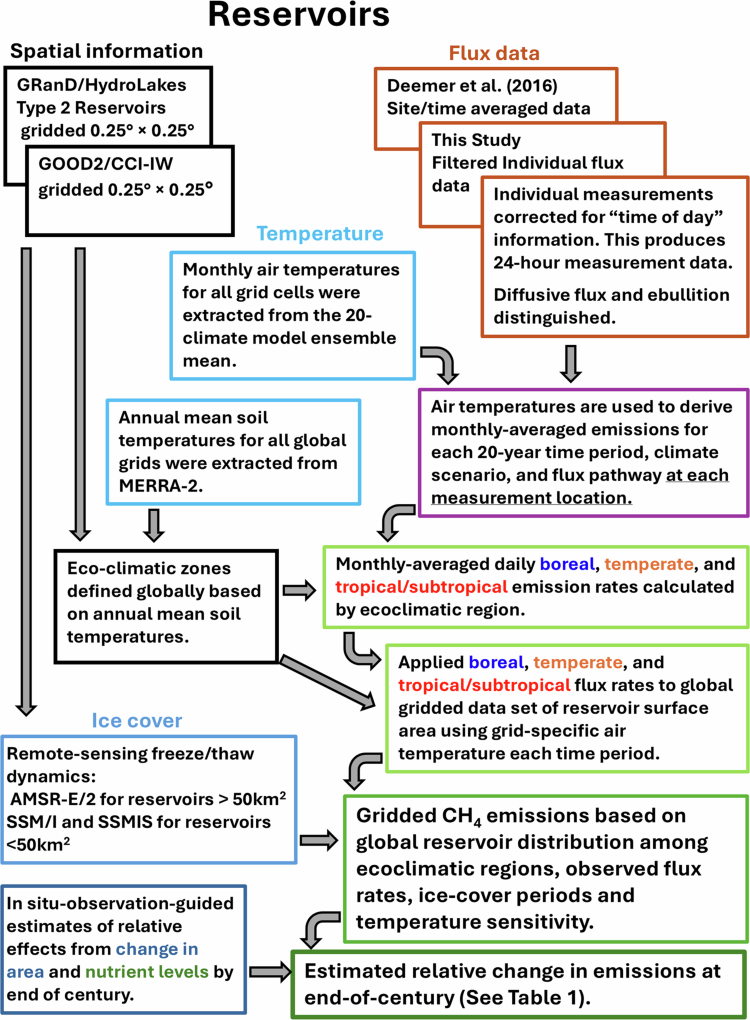
Workflow for the reservoir emissions. Extended Data Fig. 4. Workflow for the reservoir emissions, illustrating the various components considered. See Methods for details.

This is a global geospatial study, integrating available relevant in situ observed emissions by different flux types with temperature sensitivity, remote-sensing observations of ice-cover phenology, diel patterns, fluxes associated with water column storage and turnover in interaction with methane oxidation, differences among ecoclimatic regions, updated information of the area of the smallest lakes and with established quantitative projections of climate, lake area and nutrient regimes over time, to assess future CH_4_ emissions from both lake and reservoir water surfaces (Extended Data Table 4 and Extended Data Figs. 3 and 4). To our knowledge, all these aspects have not been integrated previously to predict future CH_4_ emissions from both lakes and reservoirs. Similarly, distinguishing the relative contribution of different influencing factors (predictors) have seemingly been rare. Comparisons with past work are therefore limited to subsets of the regions and lake types, lake size categories, flux types or a few of the flux predictor variables as explained for each study below. A recent regional modelling study of lake CH_4_ emissions upon permafrost thaw in the taiga plains, based on studies of 20 lakes, indicated increases of 31–121% from the Representative Concentration Pathway (RCP) climate scenarios RCP2.6 and 4.5, respectively^34^. There are also estimates of a future global lake CH_4_ production increase of 13–40% (ref. ^38^) and a 51% increase in ebullition from a warming of 4 °C (ref. ^18^). Diffusive fluxes of Finnish lakes have been estimated to increase by 26–59% until the 2090s due to a prolonged ice-free period^22^. A 1-D process-based lake model originally designed for arctic lakes, using data from 60 lakes, indicated lake CH_4_ emissions increases of 58–86% under the RCP8.5 scenario, albeit suggesting much lower absolute fluxes^12^. Studies of three subarctic lakes have led to an estimated increase in mean annual ebullition by 38% during the half-century period from 2009–2013 until 2040–2079^21^. Cumulative reservoir open-water CH_4_ emissions have been estimated to increase by 8 and 30% for diffusive flux and ebullition, respectively, from 2010 to 2040^33^. Despite the considerable differences in approaches, extent and environmental driver features considered, all studies agree on the direction, that is, that emissions of CH_4_ from lakes and reservoirs will probably increase considerably in the future. Our result, that the temperature effects are dominating (Table 1) explains why the direction of the future development is consistent. The disagreements in magnitudes are expected given the differences in scope and data support for the different studies. For the future, a greater consistency among studies in considered system types, flux types, spatio-temporal extent and environmental predictor variables would be beneficial. This would improve our underlying understanding of lake and reservoir CH_4_ emissions and allow systematic model intercomparisons, which are valuable for evaluating and constraining predictive models. In addition, it seems important that future models include not only climate and temperature effects but also effects from other potential predictors. For example, as the projections of future surface water area and nutrient loads will progress, these factors deserve more attention. They are complex to predict at high resolution and may develop in less predictable ways over space and time because of dependency on technological development and policy. For example, both lake and reservoir fluxes may be influenced by changes in agricultural practices which influence nutrient load and water use for irrigation. Hydropower reservoir area and distribution are influenced by energy policies and the cost of alternative energy sources.

### Outlook and implications for the global CH_4_ budget

Future climate effects on CH_4_ emissions have been discussed conceptually but have rarely been quantified globally and are thereby poorly constrained^39,40^. Our results of a 24–91% or 14–53 Tg CH_4_ yr^−1^ increase from present day to 2080–2099 for lakes and reservoirs depending on the climate scenario (Table 1), represent an integration of available process knowledge and in situ observations to assess possible future emissions on a global scale. For comparison with global wetlands, a data-driven integration of numerous studies estimated a climate-induced increase of wetland CH_4_ emissions of 20–60% or 14–90 Tg CH_4_ yr^−1^ (RCP2.6 to RCP8.5 scenarios)^41^. We note consistent predictions for lakes, reservoirs (present study) and wetlands^41^ in having highest relative emissions increases in boreal to arctic latitudes but high absolute emissions increases also at lower latitudes. This highlights the importance of climate effects at all latitudes.

Clearly, substantial climate effects are to be expected in natural CH_4_-emitting environments. In the business-as-usual ‘fossil-fuelled’ scenario (SSP5-8.5), the combined climate effects on lakes and reservoirs (this work) and wetlands^41^, together constituting ~10% of the continental area^7,8,42^, are likely to increase total global CH_4_ emissions by > 25% by the end of the century. Such climate effects on previously natural emissions indicate that future climate change may become more dramatic and challenging for societies than expected.

The substantial differences between the climate change scenarios illustrate that the climate effect on CH_4_ emissions from lakes and reservoirs will be highly dependent on the anthropogenic GHG emissions. Hence, urgent mitigation of anthropogenic GHG emissions is not only vital per se but also has a critical extra benefit by mitigating future positive feedbacks linked to lake and reservoir CH_4_ emissions.

## Methods

We developed a data-driven, globally gridded approach to estimate climate-driven changes in lake and reservoir CH_4_ emissions from temperature change and change in the ice-free season length under four climate scenarios for the time periods of 2000–2019 (present), 2020–2039, 2040–2059, 2060–2079 and 2080–2099 (end of the century). This model considered the major open-water CH_4_ flux types and multiple factors affecting them (Extended Data Table 4). The results from the model were combined with estimated effects on fluxes from projected changes in water surface area and nutrient levels of lakes and reservoirs (phosphorous used as proxy for biological productivity). General workflows are provided in Extended Data Figs. 3 and 4.

### Data-driven modelling for flux predictions

A previous static model for estimating contemporary CH_4_ emissions^7,8^ was further developed and extended to enable predictive capacity. The previous model was restructured to add a time dimension and allowing temporal changes in e.g. temperatures and ice-free period length under future climate scenarios (Shared Socioeconomic Pathways SSP1-2.6, SSP2-4.5, SSP3-7.0 and SSP5-8.5^43^). Climate model temperature inputs for each grid and time periods were consistently used—including the present time period (2000–2019; used as baseline). The temperature-response calculations were extended to cover both seasons and years. Climate effects on projected deep-water temperatures^38^ were incorporated. Additional aspects of uncertainty associated with predictions were added. The input CH_4_ flux data were extended to include more recent in situ CH_4_ flux observations. The model was driven by in situ observed relationships between temperature or ice-free water area over time and direct in situ flux measurements, thereby integrating net influences of the numerous complex physical, chemical and biological processes affecting CH_4_ production, oxidation and transport, without a need of knowing process-specific parameterization. Overall, we aimed for (1) maximum transparency and simplicity, along with minimum sensitivity to (2) limitations in the understanding of underlying processes and their interactions and (3) to challenges related to access to, or uncertainty of, accessory driver data. This strategy was motivated by the scarcity of local in situ data to validate all processes involved at the spatio-temporal scale needed and by the lack of time series for lake and reservoir CH_4_ emissions, which prevented traditional validation of predictive models via hindcasting. Importantly, the selected approach reduces the risk of unpredictable model behaviour based on unexpected nonlinear process interactions (further discussed below). Key parts of this model were derived from in situ observations and the general assumption that these observations collectively are representative of the ecoclimatic region and the respective lake types. An overview of all the factors being considered is available in Extended Data Table 4. The different parts of the model are described below.

#### CH_4_ flux data

Starting from available databases, the original sources providing in situ observations of lake and reservoir CH_4_ fluxes were revisited to confirm correctness of values and collect more extensive original accessory information on measurement methods, measurement times (month and time of day) and durations, water or air temperatures during each measurement, coordinates, flux type and ecoclimatic region or lake type^6–8,10,23,44^. Some new data from data-scarce regions were added specifically for this study (for example, data from Africa^45^). Altogether data from 606 individual lakes and 161 reservoirs were used. As a part of data quality control, only data from peer-reviewed publications were accepted.

#### Ecoclimatic regions, lake types and water surface area

The spatial definitions of tropical–subtropical, temperate and boreal–arctic regions were derived from annually averaged soil temperature from the Modern-Era Retrospective analysis for Research and Applications, Version 2 (MERRA-2)^46^ as described in detail previously^7,8,20^. Within the boreal–arctic region, lakes were divided into several classes based on datasets of distributions and types of permafrost, soil organic carbon content and ground-ice coverage (thermokarst, glacial/postglacial, peat pond, organic and other boreal)^20^. Geospatial area distributions of lakes and reservoirs were derived from Messager et al. (2016)^47^ for water body area > 0.1 km^2^. Smaller water bodies ( < 0.1 km^2^) were estimated from visually verified BWALD boreal–arctic small lake data^48^ as follows: BWALD data were used to derive ratios of small ( < 0.1 km^2^) to mid-size (0.1–10 km^2^) lake areas for peatland lakes (ratio of 0.86), yedoma lakes (0.82) and glacial lakes (0.24). The mean of peatland and yedoma lake ratios (0.84) was used to estimate small lake areas from mid-size lake area for our thermokarst, peat pond and organic lake categories. The glacial lake ratio was used for our glacial/postglacial lake category, and the grand mean ratio (0.54) was used to estimate small lake area in temperate and tropical–subtropical regions. The inland water areas were distributed in grids of 0.25° × 0.25°.

The water area data products used^47–49^ are remote-sensing-verified and for BWALD boreal–arctic regions also visually verified versus Google Earth satellite imagery^48^. The total lake area used was 3,099 × 10^3^ km^2^ and the area of lakes < 0.1 km^2^ had a global coverage of 405 × 10^3^ km^2^. Other area estimates of lakes < 0.1 km^2^ are up to almost 3–4 times greater (1,300 × 10^3 ^km^2^) (ref. ^9^). This highlights the importance of constraining the water area before it can be adequately considered in spatially explicit predictions. This is a reason why water area change effects in this study were considered as a proportional change factor outside the gridded model. Small and shallow water bodies are characterized by proportionally greater emissions than larger systems^18,19,50,51^ and the area estimate of the smallest water bodies can be important for absolute flux values (although less so for relative flux change). The total contemporary reservoir area used was 297 × 10^3^ km^2^ (ref. ^7^), which is slightly smaller than recent estimates from 2010 to 2020 of 315–348 × 10^3^ km^2^ (ref. ^33^).

#### Temperature predictions from climate change scenarios

For each grid cell, mean monthly near-surface (2 metres above ground level) air temperatures for the periods of 2000–2019, 2020–2039, 2040–2059, 2060–2079 and 2080–2099 under the Intergovernmental Panel on Climate Change (IPCC) Assessment Report 6^27^ climate scenarios SSP1-2.6, SSP2-4.5, SSP3-7.0 and SSP5-8.5 were derived from an aggregated ensemble comprising 20 climate models (Extended Data Table 5). Monthly mean temperatures for the time periods and all SSP scenarios used in this study were extracted from the ensemble model and used as CH_4_ flux model input. Monthly near-surface air temperature data from each model were linearly interpolated from their native spatial resolution to 0.25° × 0.25° before producing the multi-model monthly mean data. Surface water and air temperatures are typically strongly correlated over 24 h to monthly periods^52–54^. Model results from monthly grid temperatures of the period of 2000–2019 were verified towards results from corresponding observation-constrained temperature predictions from the Modern-Era Retrospective analysis for Research and Applications, Version 2 (MERRA-2)^46^ and resulting grid-specific CH_4_ fluxes deviated < ± 2.5%. This indicated a general consistency among modelled and observed temperatures in terms of effects on CH_4_ fluxes.

#### Ice-cover phenology

Spatio-temporal daily lake and reservoir freeze/thaw times for the contemporary time period were derived using satellite microwave observations of ice-cover phenology^7,8,20^. To predict future lake and reservoir ice-cover periods due to changing temperature, a second-order polynomial fit between present-day ice-free season length and 2-metre MERRA-2 air temperature was derived ($f\left(x\right)=a{x}^{2}+{bx}+c$; where $x$ is the 2-metre air temperatures; $a=$ 0.0522, ${b}=$ −21.38, $c=$ 2,109; *R*^2^ = 0.81). This fit was then applied using future SSP climate scenario 2-metre air temperatures to calculate the duration of ice cover and ice-free periods in the future for each grid and climate scenario.

#### Ebullition and diffusive flux

Open-water CH_4_ flux data not already measured over full 24-h cycles were corrected for diel variability to consistently represent 24-h fluxes as described in detail previously^55^. Field-observation-derived temperature–CH_4_-flux relationships from the literature, combined with information about the fluxes and temperatures during months of measurements, were used to estimate ice-free monthly fluxes at each measurement location. The emissions–temperature relationship was based on data listed in Extended Data Table 3. Briefly, the generic equation

1 $$\,{E}_{{{T}}}={E}_{20}\times {\theta }^{(T-20)},$$

where ${E}_{{{T}}}$ represents the emissions rate (mg CH_4_ m^−2^ d^−1^) at the surface temperature *T* (°C), ${E}_{20}$ is the emissions rate at 20 °C and *θ* represents the temperature sensitivity factor. For predictions into the future, the temperature sensitivity was applied only to ebullition due to the absence of a clear temperature relationship for diffusive flux in the most comprehensive intercontinental study^18^, which is consistent with the complex interactions among many processes regulating diffusive flux^13^. Ebullition on the other hand seems more directly linked to sediment CH_4_ production, and there is strong empirical support of its temperature sensitivity across the world^17–19,21,56–62^. A few datasets indicate that the temperature response seems general and valid both within and among years and among locations up to 45 °C (refs. ^17–19,21,62^) (Extended Data Table 3). Importantly, because the direct temperature response only regards ebullition in our predictions, the future diffusive flux is therefore only affected by climate change in the model via the length of the ice-free season. If diffusive flux is also influenced in a direct way by increased future temperatures, which some studies indicate^19,63^, our results are conservative and expected climate effects may be even greater.

By applying equation (1), the relative emission rates for each month (the proportion of yearly flux estimated to occur each ice-free month) were derived based on the monthly mean temperatures for each grid^7,8^, in turn allowing estimation of each monthly flux from available measurements. The temperature–flux relationship was used to generate extrapolated mean monthly fluxes scaled to the grid-specific temperatures as exemplified by equation (2) and equation (3) for cases where annually averaged or single month daily flux rates, respectively, were available.

2 $${E}_{{{N}}}=\left(\frac{{E}_{20}\times {\theta }^{({T}_{{{N}}}-20)}}{{\sum }_{{{N}}=1}^{12}{E}_{20}\times {\theta }^{({T}_{{{N}}}-20)}}\right)\times {E}_{{{Y}}}\times {N}_{\max }$$

3 $${E}_{{{N}}}={E}_{{{M}}}\times \frac{\left(\frac{{E}_{20}\times {\theta }^{({T}_{{{N}}}-20)}}{{\sum }_{{{N}}=1}^{12}{E}_{20}\times {\theta }^{({T}_{{{N}}}-20)}}\right)}{\left(\frac{{E}_{20}\times {\theta }^{({T}_{{{M}}}-20)}}{{\sum }_{{{N}}=1}^{12}{E}_{20}\times {\theta }^{({T}_{{{M}}}-20)}}\right)}$$

*E*_*N*_ represents the calculated monthly averaged daily emissions rate (mg CH_4_ m^−2^ d^−1^) for each month (*N* is the month number; January to December = 1 to 12) at the surface temperature *T*_*N*_ (°C) for each month, *E*_20_ is the emissions rate at 20 °C, *θ* represents the temperature sensitivity factor and *E*_*Y*_ is the annual-averaged daily emissions rate and *N*_max_ = 12. *E*_*M*_ is the known monthly emissions rate and *T*_*M*_ is the air temperature for the month of the known emissions rate. These equations are extended for transparency and because *E*_20_ cancels out, only *E*_*Y*_ or *E*_*M*_, *T*_*N*_, *T*_*M*_ and *θ* are needed as input variables.

For rescaling the temperature effect on fluxes to each future time period, the temperature-response equation was applied to rescale monthly fluxes from each grid cell to the predicted mean temperature of the corresponding months under each SSP climate scenario and time period.

The mean temperature sensitivity factor in equation (1), *θ* was 1.18 based on in situ flux temperature sensitivity studies in specific systems (Extended Data Table 3). To constrain *θ* further, we used seasonal temperature variability in the full dataset and fitted *θ* to minimize the deviation between predicted and observed mean monthly contemporary ebullition in the temperate and boreal–arctic regions (best fit at *θ* = 1.15 and 1.22, respectively; Fig. 5). Only ebullition was considered because the predicted future direct temperature response via *θ* only regarded ebullition as described above. The lack of distinct temperature seasonality at lower latitudes prevented this type of *θ* optimization and instead the mean *θ* value for tropical–subtropical in situ temperature sensitivity studies was used (*θ* = 1.09), being close to the minimum observations of 1.08 (Extended Data Table 3) and reducing the risk of overestimating the temperature sensitivity at these latitudes, contributing to making the results conservative.

Notably, the temperature sensitivity relationships derived using in situ observations represent the total integrated temperature influence on emissions via both biochemical reactions rates and substrate supply rates (via temperature effects on biological productivity) regarding CH_4_ production and CH_4_ oxidation and on CH_4_ transport processes that are correlated with temperature.

#### Episodic high-latitude CH_4_ fluxes upon ice-out and water column turnover events

The grid-specific water freeze/thaw prediction affects open-water diffusive flux and ebullition via the ice-free season length. This seasonality also affects fluxes from accumulated CH_4_ under ice upon ice melt and spring water column turnover and from accumulated CH_4_ during summer stratification upon fall water column turnover^8,64^. To include such effects, we assumed that sediment CH_4_ production and water column accumulation could be estimated from ebullition rates scaled to bottom water temperatures of 5 °C under present climate to 7 °C by the end of century^38^. The accumulation was initiated after a 60 ± 15-day lag phase as observed in situ^65,66^. A substantial fraction of the accumulated CH_4_ is considered to be oxidized in the water before being emitted (75–89%; range used in uncertainty analysis)^19,66–75^. Equations (4) and (5) below were used.

4 $${E}_{\mathrm{IS}}=\frac{({D}_{\mathrm{ICE}}-{L}_{{\rm{T}}})\times {E}_{{\rm{m}}}}{{L}_{{\rm{b}}}}\times (1-{F}_{\mathrm{mox}}),$$

where *E*_IS_ represents the calculated combined daily ice out and spring turnover emissions rate (mg CH_4_ m^−2^ d^−1^), *D*_ICE_ is the length of the ice-cover season for each model grid, *L*_T_ is the accumulation lag phase, *E*_m_ is the lake type-specific annual-averaged emissions rate, *L*_b_ is the burst length of ice-out emissions (assumed to occur for 14 days around ice melt), and *F*_mox_ is the daily oxidation fraction.

5 $${E}_{{\rm{F}}}=\frac{(365-{D}_{\mathrm{ICE}}-{L}_{{\rm{T}}})\times {E}_{{\rm{m}}}}{{L}_{{\rm{b}}}}\times (1-{F}_{\mathrm{mox}}),$$

where *E*_F_ represents the calculated daily fall turnover emissions rate (mg CH_4_ m^−2^ d^−1^) and other factors as above. *L*_b_ for fall turnover was assumed to be 7 days.

#### Scaling of large lake fluxes

Presently available flux data from large lakes are rare but indicates that emissions per m^2^ from lakes ≥ 5,000 km^2^ are in the range of 0–25% of emissions from smaller lakes^2^. Therefore, a corresponding scaling factor for such large lakes were used (average 0.1 but the full range of 0–0.25 used in uncertainty analyses).

#### Reservoir emissions pathways

For reservoirs, the emissions from turbine degassing and downstream riverine fluxes were not considered. Such fluxes have been estimated to amount for more than one-third of reservoir emissions^33^ but are linked to reservoir design and whether the turbine inlet is positioned in anoxic water with large amounts of accumulated CH_4_ or in oxic water with little CH_4_. Given the primary dependency of technical design, this flux was not considered to be sensitive to environmental change in the same way as other targeted flux pathways.

### Effects of future water area changes on fluxes

Whereas the global lake water storage in the largest lakes has declined over the period of 1992–2020^76^, the total lake area has increased 0.71% from 1988 to 2015^77^. This area increase was linked primarily to smaller lakes, which also emit more CH_4_ per area unit^50^. Whereas it is difficult to predict lake area by the end of the century, a continued increase seems reasonable because of projections of increased precipitation in already humid lake-rich regions^78^. We estimated the maximum lake area increase by the end of the century from linear projection of the recent trend^77^ resulting in a 2.6% increase in lake area. However, future precipitation may be irregular, and some areas predicted to get increased annual mean precipitation may still have long dry summer periods, making a smaller change in lake area possible. We therefore also included half the extrapolated increase, 1.3%, by the end of the century as a realistic low-end global scenario and applied an in-between estimated change factor of 1.02 using 1.013 and 1.026 as uncertainty range ( ± 1 SD) to estimate possible contribution of lake area change to future fluxes from non-thermokarst lakes. It is premature to try to distinguish the geospatial distribution of this effect at present given the uncertain area distribution of lakes < 0.1 km^2^, and overall results indicate that the temperature-related effects are much greater than the possible area change effects (Table 1). Hence, the change factors for effects of future water area were applied by ecoclimatic region and lake types (Table 1).

The future distribution of thermokarst lakes, formed from thawing of permafrost and having high areal CH_4_ emissions, is debated. Whereas models predict increased thermokarst abundance and area in the near future, remote sensing has revealed that thermokarst lake area has decreased between 2000 and 2021 in spite of rapidly ongoing permafrost thaw^79^. These actual observations showed an area decrease rate of 0.0007 to 0.0013 yr^−1^, which would correspond to a 7.3–10.6% loss of thermokarst lake area by the end of the century. A mean change factor of 0.91 (9% loss) was used to modulate the model predictions for thermokarst lakes and the full range was used as an estimate of ±1 SD in the uncertainty analysis.

Reservoir area is likely to increase considerably if the plans for new reservoirs are realized. Recent increases in reservoir area revealed by remote sensing during 1988–2015 corresponded to 9.5% resulting in a 35% increase by end of the century if extrapolated^77^. Half of this area increase was arbitrarily considered as a low-end estimate (17.5% increase), assuming some plans may be abandoned as alternative power sources are becoming more cost effective. We used a mean change factor between these estimates (1.26) when estimating effects of end-of-century reservoir area while the range of 17.5 to 35% increase was used as a ± 1 SD estimate in the uncertainty analysis. This is comparable with another recent study estimating reservoir areas to increase by 21% between 2010 and 2040^33^ probably followed by gradual stabilization as suitable new sites for reservoir construction becomes increasingly depleted and solar and wind power becomes more competitive.

### Possible influence on fluxes from changes in nutrient levels

Increased nutrient levels have been shown to stimulate CH_4_ fluxes via increased primary productivity^80^ and it has been suggested that eutrophication will increase future inland water CH_4_ emissions^81^. To estimate the magnitude of the nutrient change effect we combined the relationship between CH_4_ emissions and total phosphorous (P) concentrations developed by Beaulieu et al. (2016; data from their Table 2)^81^ and projected future river P export (proxy for catchment nutrient load) under different SSP climate scenarios until 2050^28^. The river P export was projected separately for the industrialized countries (IC; largely corresponding to temperate, boreal, subarctic, and arctic regions in our regionalization), and Brazil, India and China (BIC) and the rest of the world (RW) which were approximated to our subtropical and tropical regions). It was concluded that river P transport will change by −18.2 to +4.5 % in the industrialized world (decrease in all but the SSP3 scenario) and by +12.0 to +36.0% in BIC + RW. We assumed increased P prices along with development of more effective fertilization practices and better sewage treatment globally by 2050, which will counteract further BIC + RW eutrophication as observed in the past trajectory for industrialized countries. We therefore used 2050 projections also for end-of-century CH_4_ emissions estimates. These P changes translate to CH_4_ flux change factors ranging from 0.90 (10% decrease) to 1.19 (19% increase) depending on ecoclimatic region and SSP climate scenario (Table 1). Notably, the predicted nutrient load effect was lower under the SSP5-8.5 scenario than under the SSP3-7.0 scenario^28^.

This assessment has two main types of uncertainties. One is the relationship between CH_4_ fluxes and P, which were derived by combining relationships between CH_4_ fluxes and chlorophyll-a concentrations and between P and chlorophyll-a^81^. This link between productivity and CH_4_ emissions is highly uncertain in terms of absolute fluxes, with *R*^2^ values in the order of 0.3 and a CH_4_ emissions 95% confidence interval being of similar magnitude as the mean CH_4_ emissions^81^. Therefore, the CH_4_ emissions versus P equation was tested with coefficients representing the low and high bounds of the 95% confidence interval. Despite large differences in absolute fluxes among models, the proportional relative effect of changing P load on fluxes (change factors in Table 1) were largely consistent and within 4% of the mean (best-estimate) model. The other type of uncertainty regards the estimated future changes in P load. This uncertainty is probably large and in the absence of uncertainty estimates in the source information, we estimated ±1 SD in our uncertainty analysis by using the full range of P change estimates across all regions and climate scenarios (that is, after applying the change factors 0.89 and 1.21 in Table 1). Thereby, we possibly overestimate the uncertainty for single regions and under single climate scenarios.

### Uncertainty estimation

The uncertainty estimates calculated for reservoir and lake emissions integrated up to 11 factors. Total uncertainty linked to individual observations ($\varepsilon$) was expressed as the integrated coefficient of variation (CV) as percent of the mean in our CH_4_ emissions estimate. The calculation for lakes propagated individual uncorrelated uncertainties from each factor including the temperature in the climate scenarios (*ε*_T_), ice-free time projections (*ε*_i_), soil temperature threshold used to separate temperate and tropical/subtropical regions *(ε*_t_*)*, diffusive and ebullitive emissions measurements (*ε*_v_), temperature sensitivity of ebullition (*ε*_*θ*_), lag time for CH_4_ accumulation for calculating ice out *(ε*_ai_*)* and fall water-column turnover flux *(ε*_af_*)*, the fraction of accumulated CH_4_ which is oxidized *(ε*_ox_*)* and emissions scaling factors applied to large lakes *(ε*_sf_*)*, the lake-area change effect including thermokarst and non-thermokarst lakes *(ε*_ar_*)* and the effect of nutrient levels change on fluxes *(ε*_n_*)* through equation (2).

6 $$\varepsilon =\sqrt{{{\varepsilon }_{{\rm{T}}}^{\,\,\,2}+\varepsilon }_{{\rm{i}}}^{\,2}+{\varepsilon }_{{\rm{t}}}^{\,2}+{\varepsilon }_{{\rm{v}}}^{\,\,2}+{\varepsilon }_{\theta }^{\,\,2}+{\varepsilon }_{\mathrm{ai}}^{\,\,\,2}+{\varepsilon }_{\mathrm{af}}^{\,\,\,2}+{\varepsilon }_{\mathrm{ox}}^{\,\,\,\,2}+{\varepsilon }_{\mathrm{sf}}^{\,\,\,2}+{\varepsilon }_{\mathrm{ar}}^{\,\,\,\,2}+{\varepsilon }_{{\rm{n}}}^{\,\,2}}$$

The same uncertainty propagation was applied for reservoirs by implementing relevant uncertainties from *ε*_T_, *ε*_i_, *ε*_t_, *ε*_v_, *ε*_*θ*_, *ε*_ar_ and *ε*_n_ in equation (6).

To compare the relative uncertainty contributions from the different factors, proportional effects on equation (6) results with all factors included, versus with each factor omitted was estimated (Fig. 5).

### Design choices and associated uncertainty and limitations

Overall, our aim was to model spatio-temporally explicit environmental change effects on CH_4_ fluxes from reservoirs and lakes based on quantitative and in situ supported information. Because long time series of in situ observed lake or reservoir CH_4_ emissions are missing, it was not possible to validate our model versus historical trends. Furthermore, because of the aim to focus on in situ observations, to minimize the dependency of many ex situ determined predictors and to avoid added uncertainty from separate predictor models, our approach was developed with inspiration from wetland models developed at stages when the lack of systematic data over time was similar to the current lake and reservoir situation^35,82,83^ and predict regional averages over 20-year time periods. This excludes the option of validation using predicted versus observed data for specific systems. Further, the need to group data by ecoclimatic regions and lake types to enhance spatial data representativity, made Monte Carlo approaches to estimate uncertainty suboptimal. The reliability of our approach therefore is linked to key tradeoffs as exemplified below:

(1) One core assumption is that the data available (after quality control and consideration of transparency and method compatibility) properly represents the different system types, ecoclimatic regions and flux types. Given that the results are generated from most of the available data that covers all ecoclimatic regions and a great variety of environmental conditions (Extended Data Fig. 2), the degree of comprehensiveness and representativity should be as high as presently possible. As for all global predictive ecosystem models, data availability and coverage are typically more restricted than desired and additional systematic observations covering the lake-rich regions will be beneficial for improving the flux estimates. Importantly, the results on relative flux changes are less sensitive to the representation bias than the absolute fluxes.

(2) Another key related aspect is that future projections were based on available quality-approved in situ observations not only for the CH_4_ fluxes but also for the predictors. For the predictor variables historic in situ verified trajectories (for example, ice cover periods, lake area and river P) were used, along with attempts to incorporate their uncertainty in the analysis as described above. Accordingly, the factors considered, and their variability among environmental conditions, integrates the in situ complexity of all the underlying processes under as many environmental conditions as possible. This yields robustness to insufficient process understanding and to effects of unexpected nonlinear process interactions.

(3) Further, the choice to constrain predictions to present in situ observations and present mechanistic understanding by relatively simple and transparent calculations, along with the averaging to grids and ecoclimatic regions, leads to both limitations and benefits. One of the limitations is a potential conservative bias because nonlinear effects from unexpected process interactions will not be predicted. Other limitations are not having as high spatio-temporal resolution predictions as in more detailed process-based models, nor taking advantage of the latest developments regarding data-driven models by machine learning. On the other hand, benefits of our approach include having the above-mentioned high robustness to unrealistic predictions from nonlinear process interactions that may otherwise progress unconstrained over time. Similarly, the risks associated with applying machine learning models outside the training data domains is reduced. The typical way to mitigate these issues in both process-based and data-driven modelling, by validation of predictions via hindcasting is not possible because of the lack of time series data. Accordingly, the transparency and simplicity of our approach is beneficial by greatly facilitating the detection of unreasonable results.

(4) Additional aspects of the choice of a grid-based approach include that the grid-specific uncertainty is dependent on how well the lakes in a specific grid reflect the regional observation data imposed on that grid via the model. A grid cell with many lakes will probably represent the regional-scale variability better than a grid cell with only one lake. This means that the uncertainty at specific grids can be higher or lower than the aggregated regional estimates, in a similar way as the uncertainty for individual years may differ from the uncertainty for the integrated 20-year time periods. Generally, the associated grid-specific spatial uncertainty generated by lake aggregation is expected to be higher at lower latitudes, south of the lake-rich landscapes influenced by the last glaciation (compare with the lake density map by grid in Fig. 2a in ref. ^8^). These areas account for a high share of both current and future emissions, highlighting the need to generate enough data to allow future evaluation of the lake aggregation effects in grid-based models. The focus on uncertainty at the regional scale in this study was to ensure that uncertainty was analysed at compatible scales for all included information (for example, Fig. 5a), incorporating the fundamental spatial uncertainty associated with the spatial integration of the original observations.

The grid-based approach also means that individual lakes and their characteristics were not considered specifically. Consequently, some lake morphometry features known to influence CH_4_ emissions, such as depth, were not considered in individual lakes and it was assumed that data from the respective ecoclimatic regions to a reasonable extent represents the associated lake characteristics. Such morphometry characteristics, including annual mean depth, could also be considered largely constant over time, supported by the small predicted change in lake area over time. This makes the estimated relative flux change less sensitive to a data bias than the absolute flux estimates. Another benefit of the grid choice is compatibility with other gridded products.

(5) The temperature sensitivity *θ* is a key variable for extrapolation among seasons and years and it yielded the highest potential uncertainty of all factors when using traditional error propagation based on SD of individual system observations (Fig. 5a). However, after regional optimization of *θ* to monthly average fluxes in our dataset, the integrated uncertainty was substantially reduced (Fig. 5b). This indicates that classical error propagation overestimated the uncertainty and that our approach was capable of predicting temperature effects at a better-than-expected accuracy given the high variability among individual flux observations. Still, we acknowledge a need for more in situ studies of CH_4_ flux temperature sensitivity at all latitudes and in all types of systems for improved future predictions.

Any temperature sensitivity model can have intrinsic features that make the associated temperature-sensitivity coefficient influenced by the absolute temperature range considered. This is important to consider when making comparisons across latitudes. We evaluated this using the standard Arrhenius equation for a theoretical process with an activation energy of 70 kJ mol^−1^ as reference case. We then calculated *θ* for this process at each temperature from 0 to 50 °C, which revealed that *θ* decreased 0.04 % per °C compared to the reference relationship. Accordingly, an increase in the mean temperature of as much as 10 °C would decrease *θ* by less than 0.5%, which is minor compared with the uncertainty margins of approximately 20% (Fig. 5b). Further, this effect was mitigated by the *θ* optimization at the temperate and boreal–arctic regions that calibrated *θ* to the regional in situ observations, and by using a low-end *θ* for tropical and subtropical latitudes.

(6) Effects of factors with large local uncertainty and being important for fluxes in specific regions, for example, ice-out and seasonal water-column turnover emissions are in general challenging to predict, and our approach can only provide regional average estimates. However, these factors contributed small shares of the total global fluxes and flux changes and thereby also probably small shares of the prediction uncertainty (Fig. 5a).

(7) There is of course also some uncertainty associated with the used climate scenarios. To their support, the contemporary (2000–2019) gridded temperatures from the ensemble climate model used was successfully validated to observations (described above in paragraph Temperature predictions from climate change scenarios). Beyond this validation, we rely on extensive past work to derive and evaluate the climate scenarios. For this study the core aspect is that they encompass the likely range of future conditions.

Overall, the core strategies behind our approach were to maximum robustness, firm connections to in situ observations, in situ verified relationships and mechanistic understanding, minimize the need for assumptions, along with transparency and simplicity, based on careful consideration of amounts and characteristics of available data. While we thereby chose a simpler approach compared to more complex alternative approaches, the strategies were successful in yielding predictions that are well supported by, and highly realistic compared to, in situ observations from widely different conditions. The methods were also designed to generate conservative predictions and avoid unconstrained overestimates. Hence, the distinct results of large climate change effects on lake and reservoir CH_4_ emissions, showing clearly how we need to change our view on natural GHG flux balances and their climate sensitivity, are more likely underestimated than the opposite, making the main conclusions highly robust.

### Reporting summary

Further information on research design is available in the Nature Portfolio Reporting Summary linked to this article.

## Supplementary information

Reporting Summary

## Figures and Tables

**Extended Data Table 1:**
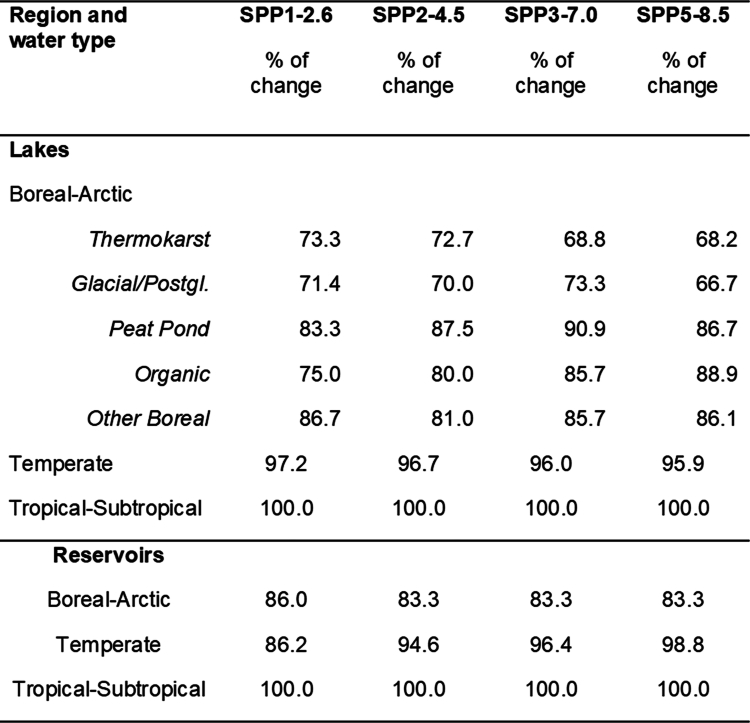
Direct temperature effects on fluxes

Percentage of the modeled change in CH_4_ emissions from lake and reservoir water surfaces that was due to direct temperature effects on fluxes (i.e., not due to change in season length) by the end of the century (2080-2099). The estimates are categorized by ecoclimatic region and water body types.

**Extended Data Table 2:**
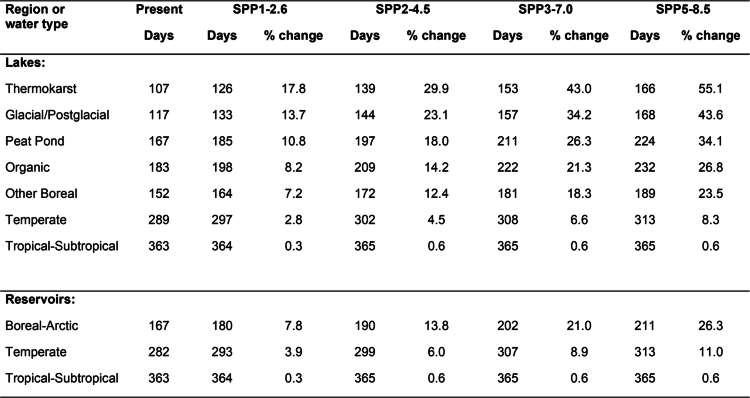
Ice-free periods under different climate scenarios

Estimated change in ice-free period lengths for lakes and reservoirs under different IPCC climate scenarios.

**Extended Data Table 3:**
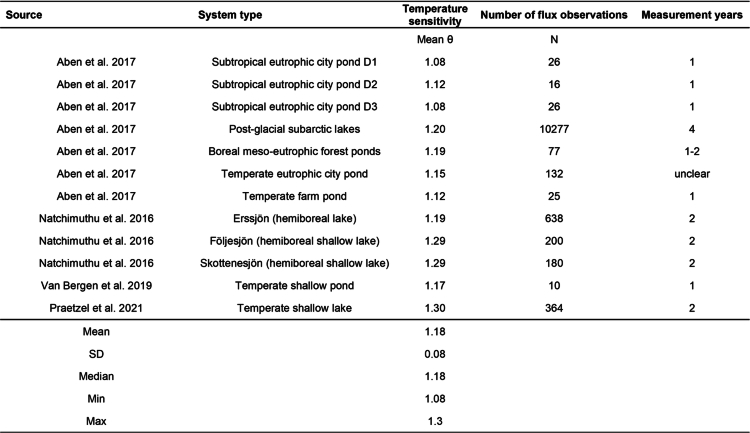
Field observations of temperature sensitivity of ebullition

Field observations of temperature sensitivity of ebullition in lentic systems reported in ways compatible with Eq. 1, ${E}_{T}=\,{E}_{20}\,\times \,{\theta }^{\left(T-20\right)\,}$, where ${E}_{T}$ represents the emission rate at the temperature *T* (°C), ${E}_{20}$ is the emission rate at 20 °C, and $\theta$ represents the temperature sensitivity factor (see Methods).

**Extended Data Table 4:**
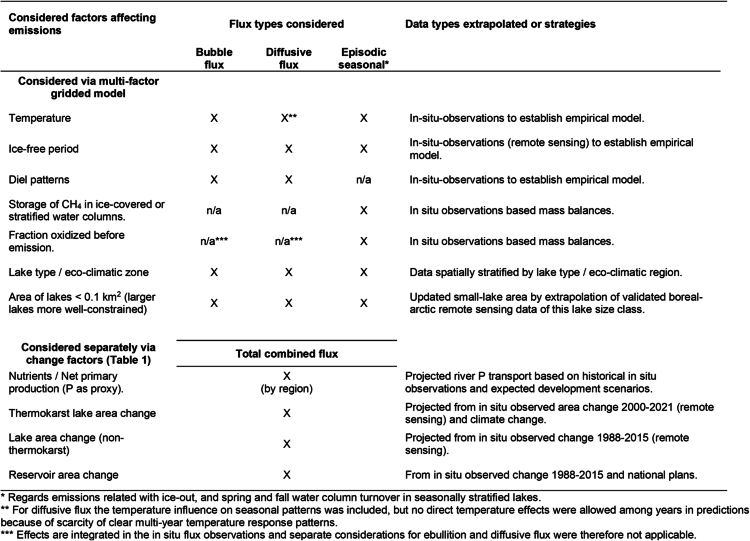
Overview of factors affecting the CH_4_ emission from open waters that were considered

Overview of factors affecting the CH_4_ emission from open waters that were considered in this study (denoted by “X”). See Methods for details and references. A main strategy was to include complex interactions of underlying processes in situ, and to minimize the number of parameters requiring estimation, fitting, and specific validation in the model. Therefore, a primarily data-driven analysis was in focus and all data used were ultimately linked to in situ observations while experimental ex situ data were not used.

**Extended Data Table 5:**
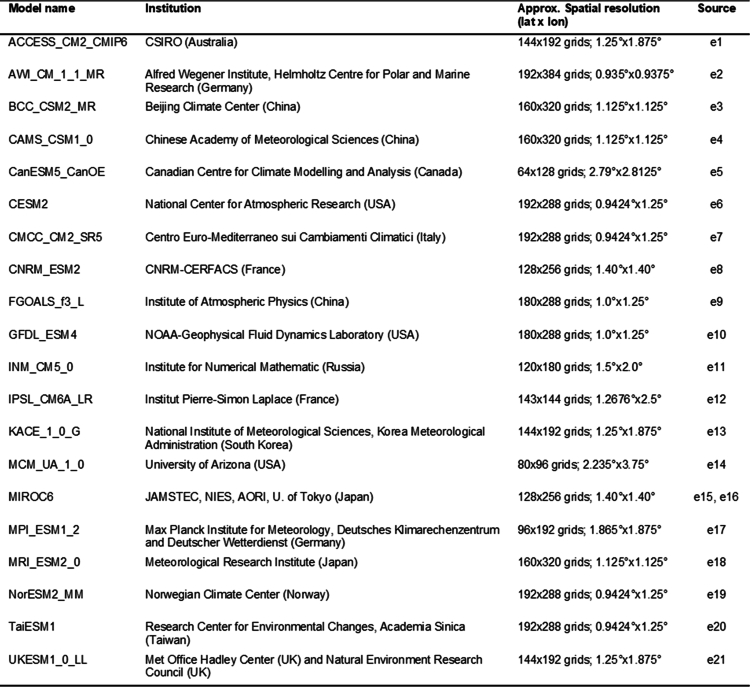
Models used to generate period ensemble climate predictions

Climate models used to generate the 20-year period ensemble climate predictions used

## Data Availability

Data used to derive the results are deposited via Zenodo at 10.5281/zenodo.17193077 (ref. ^84^).

## Code availability

The Matlab code used to derive the results is available via Zenodo at 10.5281/zenodo.17193077 (ref. ^84^).
